# Free and simple GIS as appropriate for health mapping in a low resource setting: a case study in eastern Indonesia

**DOI:** 10.1186/1476-072X-10-15

**Published:** 2011-02-25

**Authors:** Rohan P Fisher, Bronwyn A Myers

**Affiliations:** 1 Charles Darwin University, Darwin, Northern Territory 0909, Australia

## Abstract

**Background:**

Despite the demonstrated utility of GIS for health applications, there are perceived problems in low resource settings: GIS software can be expensive and complex; input data are often of low quality. This study aimed to test the appropriateness of new, inexpensive and simple GIS tools in poorly resourced areas of a developing country. GIS applications were trialled in pilot studies based on mapping of health resources and health indicators at the clinic and district level in the predominantly rural province of Nusa Tenggara Timur in eastern Indonesia. The pilot applications were (i) rapid field collection of health infrastructure data using a GPS enabled PDA, (ii) mapping health indicator data using open source GIS software, and (iii) service availability mapping using a free modelling tool.

**Results:**

Through contextualised training, district and clinic staff acquired skills in spatial analysis and visualisation and, six months after the pilot studies, they were using these skills for advocacy in the planning process, to inform the allocation of some health resources, and to evaluate some public health initiatives.

**Conclusions:**

We demonstrated that GIS can be a useful and inexpensive tool for the decentralisation of health data analysis to low resource settings through the use of free and simple software, locally relevant training materials and by providing data collection tools to ensure data reliability.

## Background

GIS is a powerful tool for improving the understanding of data through visualisation and analysis, and is being increasingly used by public health professionals for planning, monitoring and surveillance [1]. Presenting data in maps can provide more insight than a table of the same data, enabling quick assessments of trends and interrelationships [2]. This capability can assist in targeting public health initiatives [3] as well as evaluating health programs and informing long term planning. Providing equitable minimum health services is a particular challenge in developing countries where health resources and transport infrastructure are often poor [4]. Access to health services is the primary determinant of utilisation of these services [5-7] and GIS tools are being increasingly used to evaluate the distribution of health resources (e.g. [7-11]).

Despite this potential, the use of GIS in developing countries is not widespread. There are perceived problems with GIS in low resource settings[12-14]; (i) GIS software can be expensive and complex, (ii) input data are often of low quality but presentation in GIS suggests veracity; and (iii) there may be a low capacity for data analysis with the danger of misinterpretation.

Various authors have described the potentially undemocratic nature of GIS and how it can exacerbate power imbalances through disenfranchising those without the skills and infrastructure required to work with the technology [15,16]. Many GIS applications in developing countries rely on either proprietary software and/or a web interface requiring internet access [17-22]. Furthermore most of these applications require specialist data base skills to set up the back end data system for display and querying which limits health GIS to those with sophisticated data management skills and access to the internet.

A common response designed to democratise GIS has been the development of web based tools to allow more decentralized access to health GIS applications [18-20,23,24]. Paradoxically, due to low levels and unequal distribution of internet access both between and within countries, the web itself is seen by many to be an undemocratic tool in the developing world preferentially empowering an urban elite and further disenfranchising the rural poor [25-27]. For example the latest figures (2010) for internet users as a percent of population show 11% for Africa and 12.3% for Indonesia, compared to 58% and 77% for Europe and North America respectively [28]. However new opportunities for the more widespread use of GIS in low resource settings are emerging out of recent developments in GIS software and associated hardware.

This study addresses issues related to the prohibitive expense and complexity in GIS by exploring the possibilities presented by free or open source GIS software not reliant on internet access, decreasing hardware costs (e.g. GIS-ready laptops less than USD400), and the increasing availability of spatial data [17,23] and spatial data collection tools (e.g. GPS enabled mobile phones). Through the introduction of a simple and inexpensive GIS in the developing context of eastern Indonesia, this study assessed whether these tools were appropriate for increasing the data analysis capabilities at local levels. Three GIS applications were implemented over a nine month period with mentoring support from Australian researchers. Training materials were tailored to the existing technical skills common in the young health department staff, and basic equipment was provided. The evaluation included an assessment of the uptake of the technology in this context and the sustainability of this uptake.

### Study Area

Eastern Indonesia faces the challenges of providing adequate and equitable health services to a largely remote, rural population. Health in the eastern Indonesian province of Nusa Tenggara Timur (NTT) is generally poor, with high incidence of malaria [29], high infant mortality rate (54/1000, compared with 44/1000 nationally), and child malnutrition averaging 39% and reaching 50% in some areas [30].

**Figure 1:**
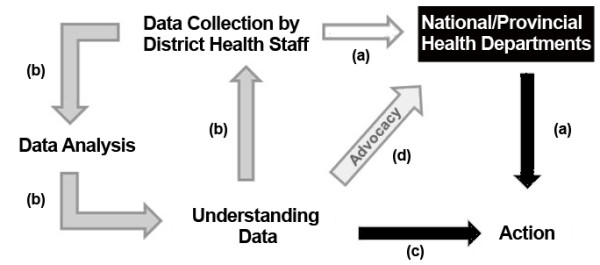
**Schematic representation of the flow of health data in eastern Indonesia**. Currently (a), district staff send health data to the provincial and national levels to inform allocation of resources back to the districts. Potentially (b), district health staff could develop the capacity to analyse and interpret data to (c) inform district level resource allocation and to (d) advocate for appropriate resource allocation from the national level.

In Indonesia, the responsibility for health service delivery is mainly at the district level. Due to decentralization since 2000, there has been devolution of budgeting and planning responsibility to the district level [31] without a corresponding provision of the base data and analysis skills required for evidence-based decision making. With the devolution of much authority and many functions to the district level, there have been disruptions to coordination and planning mechanisms, and difficulty in providing levels of public service delivery as high as those before decentralisation [32]. Currently, district and subdistrict health officers collect and collate health data for analysis at the provincial or national level (Figure 1a). However, with limited involvement in the subsequent processes of analysis, there is little sense of ownership of the data and data quality is often poor. Improving the quality of health data is a goal of a range of projects in Indonesia, yet there is little incentive at the local level to assure data quality when there is a limited understanding of how these data are to be used.

Increasing the capacity of district health officers to conduct data analysis could improve their understanding of the data they collect (Figure 1b) and so encourage the collection of more reliable data. District staff could base their health programs (Figure 1c) on the collected data and be empowered to advocate for targeted programs to address gaps in existing health services (Figure 1d).

#### Site Locations

**Figure 2:**
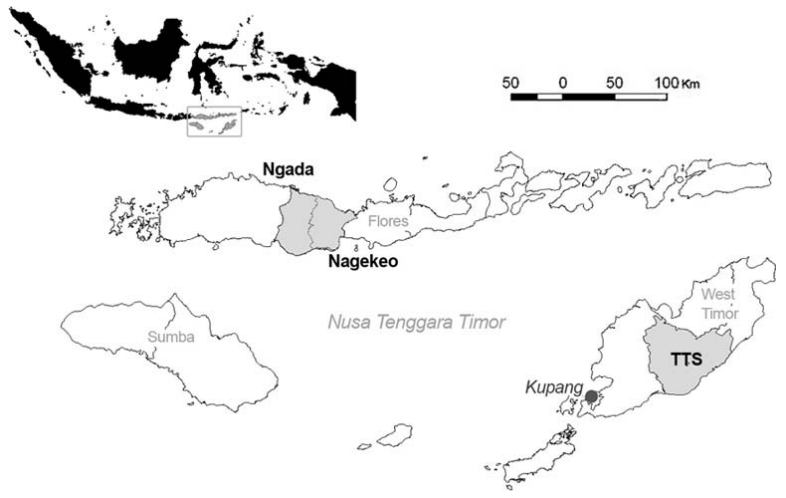
**Location of study districts (Ngada, Nagekeo, Timor Tengah Selatan (South Central Timor, TTS) in the eastern Indonesian province of Nusa Tenggara Timur**. The provincial capital, is Kupang, in West Timor.

The trial sites were located in three districts (*kabupaten*) in the eastern Indonesian province of Nusa Tenggara Timur (NTT), one of the five poorest provinces in Indonesia [33]. These districts were South Central Timor (Timor Tengah Selatan, TTS), in West Timor, and Ngada and Nagekeo, in central Flores (Figure 2). Prior to 2005 Nagekeo was part of Ngada district.

#### The health system in Nusa Tenggara Timur

In 2009, Nusa Tenggara Timur province (NTT) had a population of 4.2 million, and health facilities included 33 hospitals, 284 clinics (*puskesmas*), and more than 10,000 other local health facilities (*pustu, polindes, poskesdes *and *posyandu*) [34]. National government funding is allocated directly to the 21 city and district health departments for establishing and operating health clinics. The provincial health department has a role in coordination of health resources (staff and infrastructure) and systems for surveillance of health indicators, however subdistricts can apply directly to the national government for funding to support health posts.

## Methods

### Geographic Information System Software

Most GIS products use only a small fraction of the functionality of expensive and complex commercial GIS packages. A broad range of visualisation techniques and queries can be performed using free simple open source (OS) software. It was assumed that staff who are able to update and graph data in spreadsheets (such as Excel) would have the competencies to view the same data spatially using OS GIS software.

**Figure 3:**
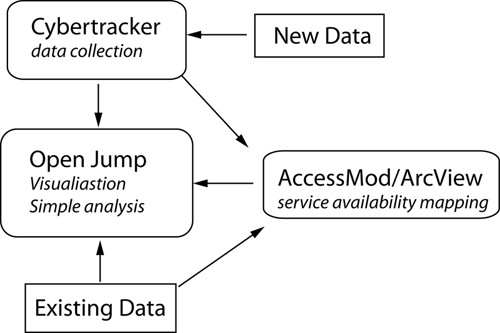
**System of data input and analysis: new reliable data collected using *Cybertracker*, new or existing data mapped and analysed in *Open Jump*, service availability modelling conducted in ArcView using the *AccessMod*^© ^extension**.

Three open source or free GIS tools were used in the three study districts: *Cybertracker* , free software for field data collection on GPS-enabled PDAs (personal digital assistant), was used to collect health infrastructure data; *Open Jump*, Java-based, open source GIS, was used to visualise health data and for simple analysis; and *AccessMod^©^*, a free extension from World Health Organisation (WHO), was used for service availability mapping (Figure 3).

Lack of reliable baseline data is commonly cited as a difficulty in implementing health GIS in the developing world [13,14,21,35]. Addressing this difficulty, training included the use of *Cybertracker *for the collection of up-to-date and accurate spatial data at the local level. Data collection using *Cybertracker *is rapid [36], includes location coordinates, and can be exported directly to a GIS format for mapping and analysis. Health mapping with *Open Jump *and service availability mapping with *AccessMod^© ^*used both new data collected using *Cybertracker *and existing data available from district health departments and clinics (Figure 3).

*Open Jump *was chosen for this study because of its intuitive interface, broad functionality and the availability of a well designed charting plug-in. OS software is of particular interest to developing countries where resources are limited and licensing costs can be prohibitive. A report commissioned by the UK government on intellectual property rights and international development, recommended that developing countries should consider OS software alternatives in their procurement policies [37]. The free and unlimited distribution of OS software and its ability to import a broad range of pre-existing data formats makes it an attractive alternative to expensive proprietary packages. Furthermore the source code is open, allowing modifications to be made locally to suit particular applications [38].

*AccessMod^© ^*is an extension to *ArcView 3.x *GIS software (ESRI) developed for the World Health Organisation to model accessibility and geographic coverage of health care infrastructure. The primary application of *AccessMod^© ^*is to produce a spatial model of accessibility as a function of travel time to health facilities. Travel time is determined by transport infrastructure, land cover (i.e. rivers, forest, grass land) and terrain. Multiple travel time estimates based on different transport modes (e.g. walking, public transport, private transport) can be produced. These data can be combined with population distribution data and health centre capacity information to produce a theoretical catchment for mapped infrastructure. Estimating access to health services as a function of travel time, rather than as linear distance, is a significant improvement particularly in rugged terrain where modes of transport vary [7,9,39-43].

Although *ArcView *is not free software, this component of the study was included to demonstrate to training participants who showed a particular aptitude for GIS how a more sophisticated level of modelling could be conducted using the collected and free spatial data. The service availability modelling was also designed to develop a broader understanding in district health departments, once basic skills were developed and data collected, of the potential of GIS with further investment. The choice, by WHO, to develop *AccessMod^© ^*for *ArcView 3.x *over other GIS platforms (e.g. *ArcGIS*) was principally motivated by its continuing widespread use and availability in developing countries [42]. We similarly choose *AccessMod^© ^*due to the common availability of *ArcView*, its free distribution and simple interface.

### Training and equipment

Each district was provided with one laptop (~USD600), one external hard drive for data backup (320 GB) and one HP iPAQ 112 PDA (~USD350) with external Bluetooth GPS (~USD50). Initially, a 3 day training workshop was conducted in the use of *Cybertracker *for field data collection and *Open Jump *for data visualisation and querying. The trainees were mostly district health department staff and some provincial and clinic staff. Their selection was based on interest in learning health mapping and likelihood that they would have opportunities to use these skills in their work. The training examples and exercises used local data from each participating district. Six months after this training, further instruction was provided to selected participants from each district in service availability mapping. Participants were given all the required software, local spatial data, and video tutorials. The tutorials provided step-by-step, screen capture instructions with Indonesian language narration, (also available at the project website [44]), enabling participants to continue self-training after the training workshop.

### Pilot Health Mapping Applications

GIS technologies were trialled in each district to guide practical implementation and test their effectiveness.

#### 1. Rapid field data collection using PDA and Cybertracker software

Before this study, there were no comprehensive audits of health infrastructure at the district level. Management of hospitals and health clinics are the responsibility of the district government, whereas other health facilities (e.g. health posts) are funded by the central government directly to the sub-district governments. This leads to the potential for the poor coordination of health resource allocation between levels of government in the absence of reliable data about existing facilities.

An audit of health infrastructure was undertaken using a simple data entry sequence in *Cybertracker *designed to collect information about the type of facility, working infrastructure (electricity, water, beds) and staff (numbers of doctors, nurses, midwives). In each district, two staff were allocated a motor bike to record these data at all health centres.

#### 2. Mapping health indicators using Open Jump software

Patient health data recorded at health clinics are reported each month, in an aggregated form, to the provincial and national health departments. Before this study, district health departments had little capacity to analyse the patient health data they collect, and as a consequence these data were rarely used to inform the allocation of health resources in their district.

Subdistrict and village administration boundaries were obtained as spatial data sets from the district planning agency (BAPPEDA). All the target districts had recently updated these data to include newly formed administrative divisions. Health data, collected at the village level and collated into annual reports were entered into the data base files (.dbf) associated with the spatial data for administrative boundaries using Microsoft Office 2003 or Open Office Calc (open source spreadsheet software). These data were then mapped in *Open Jump *using colour themeing and charting tools.

#### 3. Service availability mapping using AccessMod^©^

Most of the rural population of NTT lives in villages, often with limited access to health facilities due to rugged topography, poor roads, limited transport and seasonal flooding. Travel time from homes to health facilities was estimated using *AccessMod^©^*, taking into account terrain and seasonal variations in access (e.g. flooding). The following variables influencing travel time were provided as spatial grids for input into *AccessMod^©^*:

(i) Slope as derived from a high resolution digital elevation model (DEM). These data were obtained as a free download from the ASTER Global Digital Elevation Model (GDEM) program, a joint initiative of the Japan's Ministry of Economy, Trade and industry (METI) and NASA [45]. Slope was categorised into five classes from flat to vertical.

(ii) Land cover was produced as a grid with four categories savanna, scrub, forest and rivers. For TTS these data were produced by classifying Landsat satellite imagery obtained as a free download from the U.S. Geological Survey [46]. In Ngada and Nagekeo landcover data were available from previous projects.

(iii)Transport infrastructure comprised road data which were classified into three categories of road quality; national, provincial and district. These data were obtained from the local department of planning.

Each cell of each grid was then allocated an average travel time based on the mode of transport to be modelled, e.g. average speed walking through a scrub cell could be 2 km/hr whilst the average speed on a district level road using public transport may be 10 km/hr. These grids were combined within *AccessMod^© ^*to produce an overall travel time grid. Using *AccessMod^© ^*this grid was then intersected with the location of health facilities collected in pilot study 1 to produce models of travel time to selected health facilities.

### Evaluation of the pilot health mapping applications

The relevance and effectiveness of the training in health mapping, as perceived by the district and clinic staff who had received the training, were assessed by surveying all the trainees using written questionnaires with closed and open questions, immediately after and six months after training.

The implementation of the pilot GIS applications were evaluated by interviewing staff and officials of the participating clinics, and districts and provincial health departments. These interviews consisted of open-ended questions designed to discover if health mapping had been used to inform resource allocation planning, or in advocating for public health programs, and if so, whether these processes were an improvement on previous practices.

## Results

The results of this study included assessments of the following:

(1) the effectiveness of brief contextualized training of district health department staff with little or no knowledge of GIS, and

(2) the use of health mapping by district and clinic health department staff to inform improved health resource allocation and for advocacy.

### (1) Effectiveness of training

Surveys of the trainees indicated that, before the training, half the trainees had no prior GIS experience. Some trainees had received training in GIS using more complex software but had not used this complex software in their work because they encountered problems and were not given follow up support. These trainees found the *Open Jump *software easier to understand and use.

'Mapping using *Open Jump *is more practical and easy than using other GIS software.' [training participant]

'*Open Jump *and *Cybertracker *are appropriate... because they are simple, easy and flexible.' [training participant]

The contextualised nature of the training, i.e. exercises and examples using data from the trainees' own districts, promoted uptake of the GIS.

**Figure 4:**
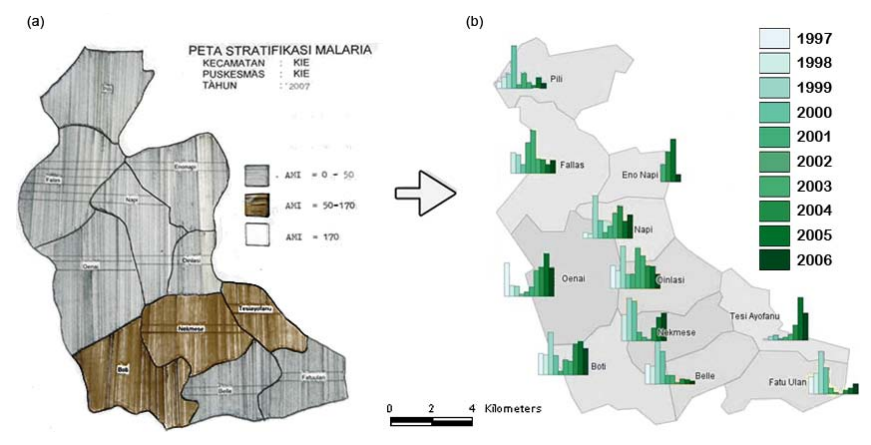
**Maps produced at a village clinic in TTS, West Timor**. (a) Hand-shaded map of number of malaria cases in the villages within the subdistrict of Kie, made before training and showing a pre-existing understanding of the value of health mapping. (b) Map, made by clinic staff after training, showing the number of malaria cases per village over a ten year period (1997-2007) from clinic data.

Before the training, some clinic staff had mapped malaria incidence by sub-district in hand-drawn maps (e.g. Figure 4a). After the training, they used *Open Jump *software to show yearly trends in malaria incidence for the same area (Figure 4b). At the completion of training, all trainees anticipated that the GIS tools would be useful in informing the targeted allocation of health resources.

'Field data collection and health mapping was very helpful especially for clinics in patient management, e.g. midwives can prioritise services for expectant mothers who are located in remote areas.' [training participant]

### (2) Use of health mapping after training

Interviews six months after the conclusion of the pilot studies showed that the trainees had applied the skills gained to new applications, to inform resource allocation and to evaluate public health programs. The GIS applications that were being used six months after the pilot GIS applications are described below.

#### Field data collection using PDA and Cybertracker software

An audit and map of all health facilities were produced by health department staff in a short time and at low cost in all target districts. For example, 176 health facilities were mapped in two districts for the cost of salaries of two staff for six weeks and the running costs of two motor bikes. Through these field audits, district health department staff became aware of some health facilities in their district that they previously did not know existed. There was a demonstrable improvement in data quality with respect to completeness of the data set and the currency of the information. One limitation of the field data collection method was that data were restricted to variables that were quickly observed. There was no opportunity to verify staffing levels, staff skills or the function of the equipment present, however the audit was used to create a framework for further investigations of infrastructure function.

'Health mapping was very useful in the analysis of relationships between health initiatives, e.g. comparisons of maps of numbers of midwives per head of population and the percentage of women receiving visits by midwives.' [training participant]

#### Visualising health infrastructure and service availability mapping informed the allocation of health resources

The maps of health infrastructure are now being used to inform the allocation of resources by the district health departments, e.g. in TTS, maps of midwives are now used in the planning of staffing allocations, and maps of clinic facilities are being used to plan the upgrading of clinics to provide basic emergency obstetric care.

Staff allocations are determined by the district administrative head (*Bupati*), usually based on recommendations from the head of the district health department. Now maps and numbers of staff per subdistrict are used to frame these recommendations.

'We use computer and PDA to know coordinate locations of health facilities to help plan the official budget each year.' [training participant]

**Figure 5:**
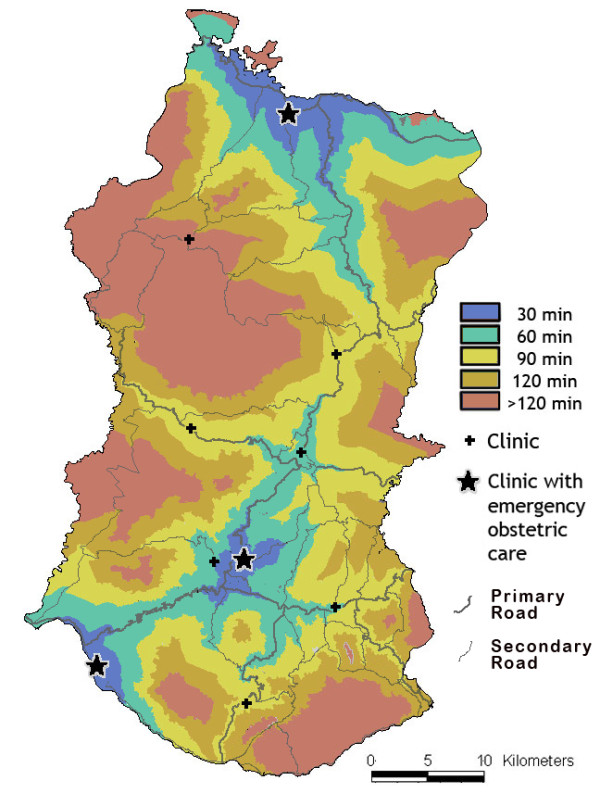
Estimated travel time of up to two hours around the clinics which provide basic emergency obstetric care and the hospital providing comprehensive emergency care in Ngada district, Flores.

Service availability mapping has been used to map the extent of estimated travel time of up to two hours (based on walking to the nearest road and travelling by motor vehicle from there) to health facilities. This type of presentation of information is informing the prioritisaton of clinics to be upgraded to provide basic emergency obstetric care and the communities in which support for emergency transport is to be developed (Figure 5).

#### Visualising maternal deaths and comparing with initiatives to encourage attendance by trained midwives at birth

At a regional health clinic in TTS district, staff have mapped maternal deaths in villages and observed that relatively high numbers were recorded for a remote village and relatively low numbers were recorded for a village where fines were imposed for births that were not attended by a trained birth assistant.

**Figure 6:**
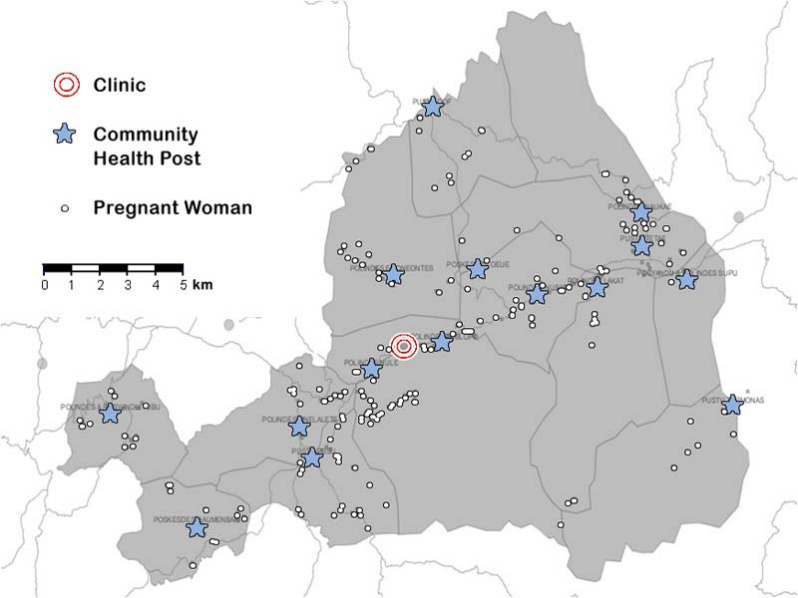
The locations of pregnant women, health facilities and major roads in one subdistrict.

Six months after the pilot studies, mapping was used to improve access to maternal health care. This was an initiative of staff at a clinic in collaboration with district health staff, in response to mapping which showed poor levels of maternal health service provision in some regions. The locations of all pregnant women in a subdistrict (n = 217) were recorded (Figure 6), along with expected delivery dates. These location data were collected by midwives whilst visiting pregnant women for routine check-ups and so required little extra work. The maps produced have been used by midwives and other clinic staff to increase efficiency of check-up schedules and to ensure clinic transport was available to provide women with access to trained birth assistance.

#### Health mapping has become a tool for advocacy

In the annual report for 2009, for the first time, the TTS health department presented data in maps instead of only in tables, charts and graphs. Maps are now also routinely used in presentations to external agencies or in professional forums as a means of clearly and succinctly explaining key health data. The head of the Provincial health department recognizes the value of maps of travel time to health infrastructure as a tool for advocating for upgrading roads in areas with poorest access to health facilities.

#### GIS tools for evaluation of public health programs

District health department staff have used GIS to evaluate the effectiveness of a child immunization program. This was an initiative of the district health dept, after the conclusion of the pilot applications project. Funding allocations to child immunization were mapped by sub-district in TTS and compared with the percentage of children who are immunized. Areas of high funding allocation and poor immunization rate were identified and are being investigated further.

## Discussion

Free and simple GIS health applications, without reliance on access to the internet, were deemed appropriate for low resource settings such as rural eastern Indonesia. District and clinic health staff demonstrated ready uptake of health GIS, and instigated and implemented a range of new health mapping applications, independent of external expertise. Maps were also being used by district health departments in NTT as tools to advocate for improved resource allocation, and in assessing the efficacy of public health programs, thus creating the potential for health mapping to inform policy [47]. Many district health staff are now offering training in health mapping to other staff members, independent of the original project. This will support the sustainability of this capacity at the district and clinic level.

We suggest that the effectiveness of the training in this pilot study was largely because simple software was used and the training was contextualized, i.e. based on exercises using data from the participants' districts. The use of local data in the training exercises prepared the trainees for applying the skills in their workplace. The step-by-step training materials, with Indonesian narration, supported continued learning after the training.

Uptake of health mapping by district and clinic staff was further enabled due to it being easily integrated into existing health information systems. These systems use spread sheets (district level) and hardcopy ledger (clinic level). Data from both these sources were easily imported into the open source mapping software (*Open Jump*) without additional data management systems.

Health data in developing countries are often of poor quality and the district staff who collect the data have little opportunity to use the data they collected to inform their own planning and resource allocation. Furthermore, data sharing between province, district and subdistrict is generally poor [48]. Rapid collection of health infrastructure data in the field over large areas empowered district health staff, for the first time, to collect reliable and complete health infrastructure data.

This study did not evaluate the quality of the patient health indicator data reported by the clinics. It is expected that timely mapping of health data by the district and clinic staff who collect and report these patient health data will highlight possible anomalies in the data set [49] and possibly lead to improvements in patient health data. Also, it is suggested that, if the staff reporting the data are also using the data to inform the public health programs they implement, there will be added incentive to ensure high quality data is collected. However even though spatial visualisation can be a tool for critical data analysis, this skill does not guarantee critical thinking. The ability to question the veracity of data, search for correlations, trends or inconsistencies are skills that need to be actively taught alongside the more technical competencies. Experience has shown that when trainees present the results of their health mapping to peers, critical thinking is encouraged both in the presenters and the audience. Although the results to date have been promising, a longer timeframe is required to evaluate the impacts of health mapping on policy and program development within district and provincial governments, and on the delivery and efficacy of health care.

Some cautionary observations were made about the specific software and hardware used. Data collection using *Cybertracker *is simple, however we found that creating a database within *Cybertracker *was limited to those with reasonable IT literacy. Some hardware problems were encountered, including difficulty maintaining a Bluetooth connection between the PDA and GPS, and a short battery life of the PDA. To ensure no field time was lost due to hardware problems, some surveys were augmented by using a standard GPS to log points, with data recording manually. Alternative PDA hardware with inbuilt GPS and a supply of batteries has largely overcome this problem.

*Open Jump *was effective for visualisation and preliminary analysis of recorded data. Some initial limitations with the *Open Jump *charting and printing functions were overcome through collaboration with the open source software engineer originally responsible for developing these components. Open source software is, by its nature, open to collaboration and sharing, and there is a large community of software developers ready to improve and refine open source tools based on intelligent feedback.

The requirement for licence software to run *AccessMod*^© ^is a significant limitation for its wider adoption although it effectively demonstrated the utility of simple modelling using free and easily collectable data. A further constraint was the limited availability of the demographic data required for comprehensive analysis. Whilst *AccessMod*^© ^provided a general indicator of coverage of health facilities, the lack of population distribution data precluded, for example, the delineation of patient catchment areas for a particular health facility. However, staff of the district health departments have local knowledge about the location of population in their district. With mapping skills developed at the district level, this local knowledge can be incorporated into the interpretation of resource allocation maps, even in the absence of precise population information.

## Conclusions

The effectiveness of free and simple GIS was demonstrated at district and clinic levels in the low resource setting of rural, eastern Indonesia. The uptake and continued use of these technologies was a demonstration of decentralised GIS, empowering those with local knowledge and public health skills. This technology was deemed appropriate because it did not require internet access, a centralized database or a need for database development or maintenance.

This low cost, internet-free approach has broad relevance in the context of the move towards the decentralisation of governance and service provision in the developing world [50,51]. We argue that whilst most of the world is still without internet access, particularly outside urban centres, dependence on web-based health GIS applications will tend to re-centralise the power of GIS and thus work against many political reforms occurring in the developing world. To truly democratize the power of health GIS, appropriate 'disconnected' technologies need to be available for the least empowered in the developing world.

## Competing interests

The authors declare that they have no competing interests.

## Authors' contributions

RF adapted GIS methods, developed and delivered locally relevant training and evaluated uptake. BM facilitated partner engagement and evaluated uptake. Both authors drafted the paper, and read and approved the final manuscript.

## References

[B1] Cromley EK McLafferty S GIS and public health 2002 The Guilford Press

[B2] Mujeeb SA Shahab S Hyder AA Geographical display of health information: study of hepatitis C infection in Karachi, Pakistan Public Health (Nature) 2000 114 5 413 11035468

[B3] Booman M Using a geographical information system to plan a malaria control programme in South Africa Bull World Health Organ 2000 78 1438 1444 PMC2560669 11196490

[B4] Peters DH Garg A Bloom G Walker DG Brieger WR Rahman MH Poverty and access to health care in developing countries Annals of the New York Academy of Sciences 2008 161 171 1136(Reducing the Impact of Poverty on Health and Human Development: Scientific Approaches) 10.1196/annals.1425.011 17954679

[B5] Müller I Smith T Mellor S Rare L Genton B The effect of distance from home on attendance at a small rural health centre in Papua New Guinea International Journal of Epidemiology 1998 27 5 878 10.1093/ije/27.5.878 9839747

[B6] Arcury TA Gesler WM Preisser JS Sherman J Spencer J Perin J The effects of geography and spatial behavior on health care utilization among the residents of a rural region Health Services Research 2005 40 1 135 156 10.1111/j.1475-6773.2005.00346.x PMC1361130 15663706

[B7] Tanser F Gijsbertsen B Herbst K Modelling and understanding primary health care accessibility and utilization in rural South Africa: An exploration using a geographical information system Social Science & Medicine 2006 63 3 691 705 10.1016/j.socscimed.2006.01.015 16574290

[B8] Tanser F Wilkinson D Spatial implications of the tuberculosis DOTS strategy in rural South Africa: a novel application of geographical information system and global positioning system technologies Tropical Medicine & International Health 1999 4 10 634 638 10.1046/j.1365-3156.1999.00469.x 10583895

[B9] Perry B Gesler W Physical access to primary health care in Andean Bolivia Social Science & Medicine 2000 50 9 1177 1188 10.1016/s0277-9536(99)00364-0 10728839

[B10] McLafferty SL GIS and health care 2003 10.1146/annurev.publhealth.24.012902.141012 12668754

[B11] Noor AM Gikandi W Hay SI Muga RO Snow RW Creating spatially defined databases for equitable health service planning in low-income countries: the example of Kenya Acta Tropica 2004 91 3 239 251 10.1016/j.actatropica.2004.05.003 PMC2673552 15246930

[B12] Thomson M Connor S O'Neill K Meert JP Environmental Information for Prediction of Epidemics Parasitology Today 2000 16 4 137 138 10.1016/s0169-4758(00)01648-3 10725896

[B13] Sipe N Dale P Challenges in using geographic information systems (GIS) to understand and control malaria in Indonesia Malaria Journal 2003 2 1 36 10.1186/1475-2875-2-36 PMC305351 14613511

[B14] Ansumana R Malanoski AP Bockarie AS Sundufu AJ Jimmy DH Bangura U Jacobsen KH Lin B Stenger DA Enabling methods for community health mapping in developing countries International Journal of Health Geographics 2010 9 1 56 10.1186/1476-072X-9-56 PMC2987786 21034454

[B15] Fox J Suryanata K Hershock P Pramono AH Fisher R, Myers B, Sanam M, Tarus V Mapping communities: The socio-ethical dimension of participatory mapping *, in *GIS Applications for Sustainable Development and Good Governance in Eastern Indonesia and Timor Leste 2009 CDU Press: Darwin 70 87

[B16] Elwood S Critical issues in participatory GIS: deconstructions, reconstructions, and new research directions Transactions in GIS 2006 10 5

[B17] Aileen C Combining Google Earth and GIS mapping technologies in a dengue surveillance system for developing countries International Journal of Health Geographics 2009 8 10.1186/1476-072X-8-49 PMC2729741 19627614

[B18] Bambang P Wayan S Gede P Donald B Spatial and multidimensional visualization of Indonesia's village health statistics International Journal of Health Geographics 2008 7 10.1186/1476-072X-7-30 PMC2494543 18544174

[B19] Bas V Open source GIS for HIV/AIDS management International Journal of Health Geographics 2008 7 10.1186/1476-072X-7-53 PMC2584066 18945338

[B20] Kamadjeu R Tolentino H Web-based public health geographic information systems for resources-constrained environment using scalable vector graphics technology: a proof of concept applied to the expanded program on immunization data International Journal of Health Geographics 2006 5 1 24 10.1186/1476-072X-5-24 PMC1523338 16749942

[B21] Lozano-Fuentes S Elizondo-Quiroga D Farfan-Ale JA Loroño-Pino MA Garcia-Rejon J Gomez-Carro S Lira-Zumbardo V Najera-Vazquez R Fernandez-Salas I Calderon-Martinez J Use of Google EarthTM to strengthen public health capacity and facilitate management of vector-borne diseases in resource-poor environments Bulletin of the World Health Organization 2008 86 718 725 10.2471/BLT.07.045880 PMC2649496 18797648

[B22] Fuller S Tracking the Global Express: New Tools Addressing Disease Threats Across the World Epidemiology 2010 21 6 769 771 10.1097/EDE.0b013e3181f56757 20924231

[B23] Cinnamon J Schuurman N Injury surveillance in low-resource settings using Geospatial and Social Web technologies International Journal of Health Geographics 2010 9 1 25 10.1186/1476-072X-9-25 PMC2881902 20497570

[B24] Vanmeulebrouk B Open source GIS for HIV/AIDS management International Journal of Health Geographics 2008 7 1 53 10.1186/1476-072X-7-53 PMC2584066 18945338

[B25] Albirini A The Internet in developing countries: A medium of economic, cultural and political domination International Journal of Education and Development using ICT 2008 4 1

[B26] Chinn MD Fairlie RW ICT use in the developing world: an analysis of differences in computer and internet penetration Review of International Economics 18 1 153 167

[B27] Hindman DB The rural-urban digital divide Journalism and Mass Communication Quarterly 2000 77 3 549 560

[B28] internet world statistics http://www.internetworldstats.com/stats.htm cited

[B29] WHO http://www.searo.who.int cited

[B30] Muslimatun SF A Brief Review on The Persistent of Food Insecurity and Malnutrition Problems in East Nusa Tenggara Province, Indonesia. *, in *IITTS Publications, Institute of Indonesia Tenggara Timur Studies 2009

[B31] (GOI), G.o.I Handbook of the Administration of Government and Regional Development 2007

[B32] Suryahadi A Monitoring S Unit ER Developing a Poverty map for Indonesia: an initiatory work in three provinces SMERU Research Institute and BPS 2003

[B33] Deasey M Hodgkin D Winahyu R Langford G Moving Forward: Oxfam International Country Analysis 2008 2008 Benchmark Consulting, Oxfam

[B34] NTT Provincial Health Information http://nttprov.go.id/cited

[B35] Linard C A high resolution spatial population database of Somalia for disease risk mapping International Journal of Health Geographics 2010 9 45 10.1186/1476-072X-9-45 PMC2949749 20840751

[B36] Beyers R Cybertracker vs ArcPad: a technical review comparing both systems (in the context of MIKE forest elephant population surveys) 2006

[B37] Câmara G Onsrud H Open-source geographic information systems software: Myths and realities 2004

[B38] Ramsey P The state of open source GIS Victoria, BC: Refractions Research Inc 2005 http://www.refractions.net/expertise/whitepapers/opensourcesurvey/survey-open-source-2007-12.pdf

[B39] Tanser FC The application of GIS technology to equitably distribute fieldworker workload in a large, rural South African health survey Tropical Medicine & International Health 2002 7 1 80 90 10.1046/j.1365-3156.2002.00825.x 11851958

[B40] Tanser F Geographical information systems (GIS) innovations for primary health care in developing countries Innovations: Technology, Governance, Globalization 2006 1 2 106 122

[B41] Schuurman N Fiedler RS Grzybowski SCW Grund D Defining rational hospital catchments for non-urban areas based on travel-time International Journal of Health Geographics 2006 5 1 43 10.1186/1476-072X-5-43 PMC1617091 17018146

[B42] Ray N Ebener S AccessMod 3.0: computing geographic coverage and accessibility to health care services using anisotropic movement of patients International Journal of Health Geographics 2008 7 1 63 10.1186/1476-072X-7-63 PMC2651127 19087277

[B43] Swan G Selvaraj S Godden D Clinical peripherality: development of a peripherality index for rural health services BMC Health Services Research 2008 8 1 23 10.1186/1472-6963-8-23 PMC2246121 18221533

[B44] Health mapping tutorial CDU http://healthpslp.cdu.edu.au/tute cited

[B45] NASA site http://asterweb.jpl.nasa.gov/gdem.asp

[B46] US geological survey http://glovis.usgs.gov cited

[B47] Tanser FC Le Sueur D The application of geographical information systems to important public health problems in Africa Int J Health Geogr 2002 1 4 10.1186/1476-072X-1-4 PMC149399 12537589

[B48] Asbanu JC Fisher R, Myers B, Sanam M, Tarus V GIS and local government in Indonesia: Experiences, difficulties and potentials in Kabupaten Timor Tengah Selatan, *in *GIS Applications for Sustainable Development and Good Governance in Eastern Indonesia and Timor Leste 2009 CDU Press: Darwin 214 223

[B49] Nobre FF Braga AL Pinheiro RS dos Santos Lopes JA GISEpi: a simple geographical information system to support public health surveillance and epidemiological investigations Comput Methods Programs Biomed 1997 53 33 45 10.1016/s0169-2607(96)01799-3 9113466

[B50] Bardhan P Decentralization of governance and development The Journal of Economic Perspectives 2002 16 4 185 205

[B51] Bardhan PK Mookherjee D Decentralization and local governance in developing countries: a comparative perspective 2006 1 The MIT Press

